# Supplementary material for: The interactome of intact mitochondria by cross-linking mass spectrometry provides evidence for coexisting respiratory supercomplexes
Source: Mol Cell Proteomics. 2017 Dec 8;17(2):216–32. doi: 10.1074/mcp.RA117.000470 (PMC5795388; doi:10.1074/mcp.RA117.000470)
Supplement: Supplemental Data [file supp_RA117.000470_133922_0_supp_23978_fzffwf.zip › spectra_annotation/mito_spectra_annotation/455-1-6-1-10-1.pdf]

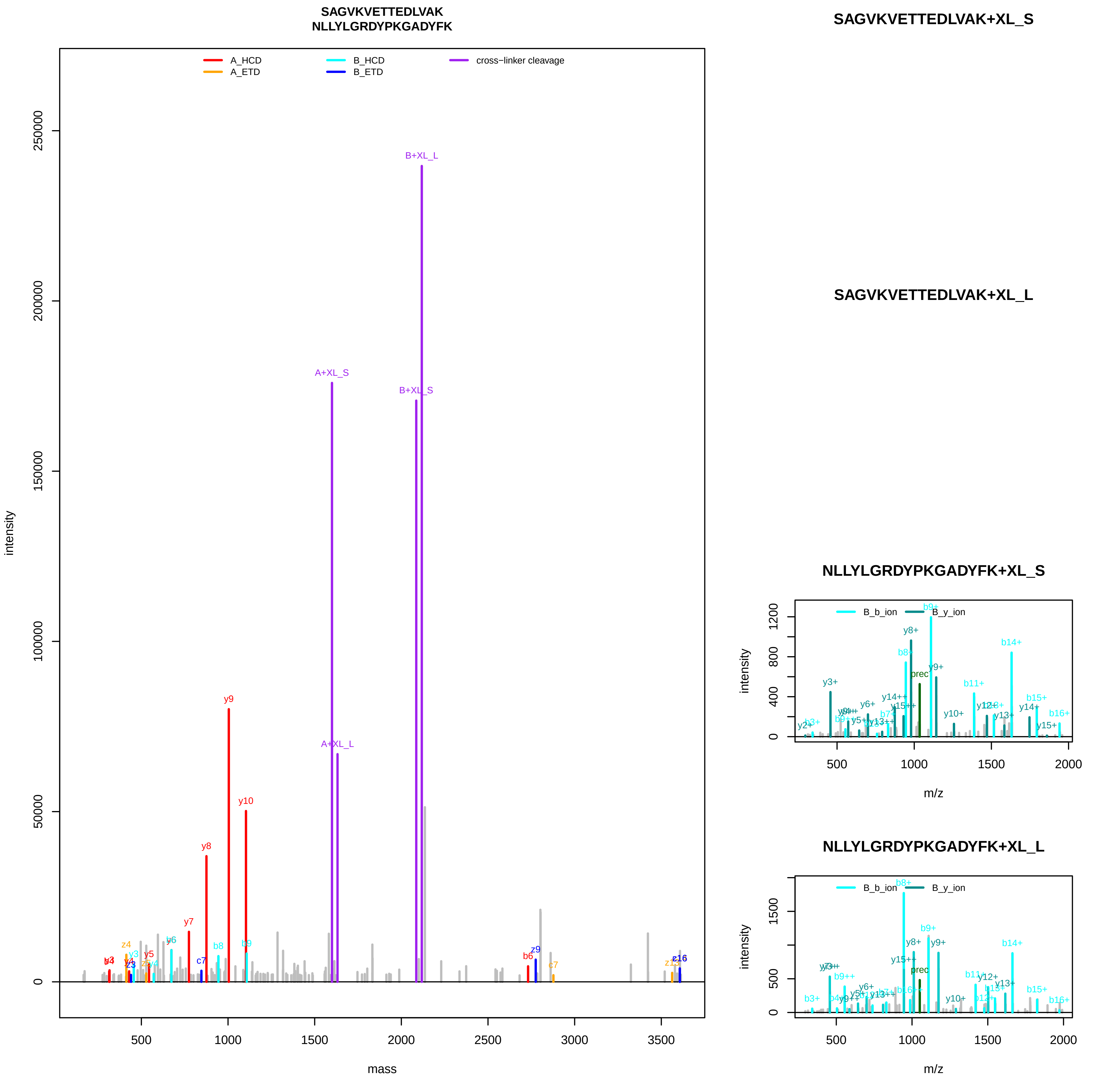
SAGVKVETTEDLVAK
NLLYLGRDYPKGADYFK
A_HCD
A_ETD
B_HCD
B_ETD
cross−linker cleavage
B+XL_L
A+XL_S
B+XL_S
A+XL_L
y9
y10
y8
y7
y5
b6
z9
c7
c16
z13
c7
b8
b9
b6
y3
z4
z3
intensity
mass
0
50000
100000
150000
200000
250000
500
1000
1500
2000
2500
3000
3500
SAGVKVETTEDLVAK+XL_S
SAGVKVETTEDLVAK+XL_L
NLLYLGRDYPKGADYFK+XL_S
B_b_ion
B_y_ion
b9+
y8+
b8+
b14+
prec
y9+
y3+
b11+
y10+
y14+
b15+
b16+
y15+
y2+
b3+
y6+
y5+
y14++
y15++
y13+
m/z
0
400
800
1200
500
1000
1500
2000
NLLYLGRDYPKGADYFK+XL_L
B_b_ion
B_y_ion
b8+
b9+
y8+
y9+
b14+
y15++
prec
b11+
y12+
y13+
b15+
b16+
y10+
b9++
b3+
y6+
y5+
m/z
intensity
0
500
1500
500
1000
1500
2000
